# CAR T-Cell Cancer Therapy Targeting Surface Cancer/Testis Antigens

**DOI:** 10.3389/fimmu.2020.01568

**Published:** 2020-09-02

**Authors:** Mie K. Jakobsen, Morten F. Gjerstorff

**Affiliations:** ^1^Department of Cancer and Inflammation Research, Institute for Molecular Medicine, University of Southern Denmark, Odense, Denmark; ^2^Department of Oncology, Odense University Hospital, Odense, Denmark; ^3^Academy of Geriatric Cancer Research (AgeCare), Odense University Hospital, Odense, Denmark

**Keywords:** CAR T cell, cancer/testis antigen, DNA methyltransferase inhibitor, cancer immunotherapy, T cell engineering

## Introduction

**Figure 1:**
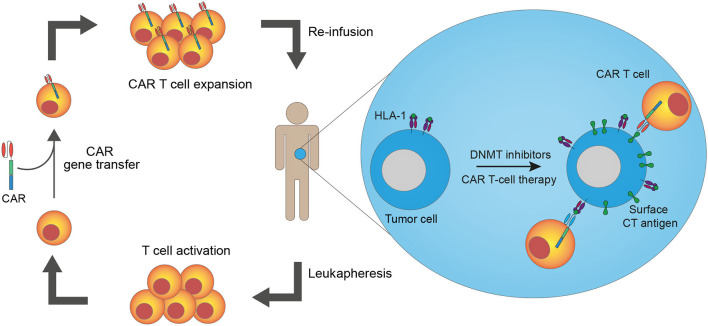
Epigenetic pre-treatment sensitizes cancer cells to cancer/testis antigen-directed CAR T-cell therapy. T cells from a patient are harvested through leukapheresis. The T cells are activated and genetically modified to express chimeric antigen receptors (CARs) *ex vivo* before they are expanded and reinfused into the patient. Pre-treatment of patients with epigenetic drugs, such as DNA methyltransferase (DNMT) inhibitors, increases cancer/testis (CT) antigen and HLA-I expression on cancer cells, leading to enhanced recognition of cancer cells by CAR T cells. Both CAR T cells made from conventional antibodies and TCR mimic antibodies (recognizing antigen peptides presented in complex with HLA-I) are available strategies.

The discovery that cancer cells are recognized and targeted by the immune system has recently increased interest in cancer immunotherapies. Currently, the most widely used immunotherapy is immune checkpoint blockade using monoclonal antibodies to block intrinsic downregulators of immunity, which are often over-expressed in cancer. Although beneficial to some patients, the majority do not respond to this treatment (1), highlighting the need for new, novel, strategies. One new approach is T-cell therapy with genetically engineered T cells to generate an effective anti-tumor immune response through *ex vivo* manipulation of the T-cell tumor-specificity. This can be accomplished by gene transfer of T-cell receptors (TCRs) or chimeric antigen receptors (CARs) into autologous T cells before reinfusing them into the patient. CAR T-cell therapy has shown excellent results in the treatment of some B-cell malignancies, but the lack of suitable antigens presents a challenge to transferring this therapy to other malignancies, including solid cancers (2). Here, we review data on cancer/testis (CT) antigens as targets for CAR T-cell therapy and present a strategy to upregulate CT antigen expression on tumor cells via epigenetic treatment to sensitize cancer cells to CAR T-cell therapy (Figure 1).

## Car T-Cell Therapy in Hematological and Solid Cancers

CAR T cells are T cells genetically engineered to express artificial receptors, called CARs, on their cell surface, facilitating enhanced recognition of specific tumor antigens and thereby killing of cancer cells (3). CARs are comprised of an extracellular antigen-recognition domain, often derived from a monoclonal antibody, and an intracellular signal-transduction domain resembling that on TCRs, containing CD3ζ and up to two costimulatory domains, such as CD28 or 4-1BB. The extracellular domain provides specificity to the CAR and directs the T cell toward cancer cells through recognition of antigens on the cell surface (4). TCRs recognize epitopes presented by HLA-I molecules on the surface of tumor cells. Since antigen processing and presentation are complex, the identification of epitopes for TCRs is laborious (5). CARs, unlike normal TCRs, recognize antigens independent of HLA-antigen processing and presentation and thereby circumvent the challenges with TCR epitope identification. Also, CARs can recognize tumor cells with downregulated HLA expression or decreased protosomal processing, which are mechanisms that contribute to antigen escape by TCR-mediated immunity (6). Correspondingly, suitable targets for CARs need to be located on the cell surface for the receptor to recognize them, which makes the potential target pool smaller compared to potential targets for TCRs (2, 7). Furthermore, the HLA-unrestricted recognition of antigens by CARs makes available CAR constructs useful for patients with all HLA subtypes and facilitates an off-the-shelf strategy (8). The safety profiles of TCR and CAR T-cell therapy are similar, but there may be important differences. On-target/off-tumor toxicity is a risk of both TCR and CAR T-cell therapy and occurs when the targeted antigen is also present on healthy tissue, but CAR or affinity-maturated TCR constructs may require lower levels of antigen expression in target cells. Similarly, the risk for off-target/off-tumor toxicities, where T cells with engineered receptors cross-react with other antigens, is enhanced with CARs or affinity-maturated TCRs or may result from the combination of native and engineered TCR chains (9, 10).

The major breakthrough for CAR T-cell therapy came with the CD19-specific CAR targeting the cardinal B-cell antigen, CD19. This therapy has shown excellent results in treating multiple B-cell malignancies, e.g., Maude et al. reported 90% complete remission in patients with relapsed or refractory ALL for up to 2 years after treatment with autologous CD19 CAR T-cell therapy (11–14). Despite the great success of the CD19 CAR, relapses due to antigen loss still occur, and new targets are needed to treat these patients (15, 16). New targets are also needed for hematological cancers that do not express CD19. The success of CAR T cells in treating hematological cancers has led to this therapy receiving a large amount of attention as a treatment for solid cancers, but so far, the clinical efficacy is limited (17–19). Where target identification and antigen loss are the major obstacles in CAR T-cell therapy of hematological cancers, multiple factors contribute to the low clinical efficacy in the treatment of solid cancers. CD19 is a linage B-cell antigen also expressed on normal B cells in the early stages of B-cell development. Targeting CD19, therefore, leads to B-cell aplasia, which is clinically manageable. Targeting linage antigens on solid tumors is not an option due to severe toxicities (2, 20). The ideal target for CAR T-cell therapy should be ubiquitously expressed on all tumor cells and be completely absent from healthy tissue to avoid complications such as on-target/off-tumor toxicities, as described above. However, these antigens have proven difficult to find. Often, a heterogeneous expression pattern within the tumor tissue and/or expression in healthy tissue limits the clinical potential of the target (21). Tumor-specific antigens include neoantigens, viral antigens, and CT antigens. CT antigens have remained unrecognized as targets for CAR T-cell therapy so far, but due to the restricted expression pattern toward tumor tissue and especially the possibility of upregulating their expression by epigenetic drugs, these antigens may represent promising candidates for CAR T-cell therapy.

In solid cancers, the CAR T cells must overcome the tumor microenvironment (TME) in order to reach the tumor cells. Initially, there is the physical barrier of the stroma that may prevent T cell entry into the tumor. Next, T cells that have successfully entered the tumor may be functionally repressed by immunosuppressive factors (like PD-L1 and CTLA-4), inhibitory cytokines (e.g., TGFβ, IL-4, and IL-10) and inhibitory cells (e.g., regulatory T cells and tumor-associated macrophages) (22).

## Cancer/Testis Antigens As Targets For Car T-Cell Therapy

CT antigens are a unique set of antigens expressed in germ cells of the testis and various malignancies of different histological origin but not in healthy somatic tissues (23). Their cancer-restricted expression pattern, along with their immunogenic properties, make CT antigens ideal targets for immunotherapy (24, 25). Methylation of promoter-regions controlling CT antigen genes is a well-known silencer of gene expression in healthy somatic tissue (26). Malignant transformation is often associated with global DNA hypomethylation, which leads to the induction of CT antigen gene expression in some tumors. However, CT antigens show a very heterogeneous expression pattern within tumors (27), most likely reflecting epigenetic variation and plasticity among tumor cells. This may give rise to immune-escape variants in the form of tumor cells not expressing the antigen, creating an obstacle when targeting CT antigens with immunotherapy. Results from us and others show that treatment with epigenetic drugs, such as DNA methyltransferase inhibitors (DNMTis), specifically upregulates CT antigen expression within tumors, thereby inducing a more ubiquitous expression pattern of the antigens (28, 29). Therefore, epigenetic treatment can be used to sensitize cancer cells for immunotherapy, such as CAR T-cell therapy, and lead to increased elimination of cancer cells (30–34). For instance, in an immunocompetent murine breast cancer model, epigenetic priming of tumors combined with adoptive transfer was demonstrated to control metastatic spread (35).

CT antigen expression is known to be mainly intracellular, which excludes many of the antigens as CAR targets, but CT antigens with a surface localization have been identified (36–38). A recent study scrutinized 3,700 different genes predicted to encode proteins located on the surface of human cells and found 22 genes with restricted expression in testis (36), many of which were upregulated in multiple hematological and solid malignancies. These genes represent highly promising targets for CAR T-cell therapy, and further investigations should be performed to elucidate the inducibility of the antigens with DNMTis, identify surface epitopes, and explore options for CAR-targeting. Other, more well-described, CT antigens have been tested as potential CAR targets. MAGE-A1 was investigated as a possible target in the treatment of lung adenocarcinoma (LUAD) after immunostaining revealed a surface epitope of the antigen (37). A MAGE-A1-specific CAR showed cytotoxic activity both *in vitro* and *in vivo*, where it was able to infiltrate MAGE-A1-positive tumors and specifically target and inhibit LUAD xenograft growth in nude mice. Further studies are ongoing to evaluate the potential of MAGE-A1-specific CAR T cells in the treatment of LUAD. Because MAGE-A1 is expressed in multiple other cancers and can be upregulated by treatment with epigenetic modulators, MAGE-A1-specific CARs could present an attractive option for the treatment of these diseases (31, 39–41). PRAME is another well-described CT antigen that has been tested as a target in several immunotherapeutic strategies. Because PRAME was previously recognized as an intracellular protein, and therefore non-targetable by traditional antibodies or CARs, a TCR mimic antibody with the same specificity as a TCR was developed (42). This molecule specifically recognized a PRAME peptide presented in complex with HLA-A2 and provided proof-of-concept that such antibodies can recognize and generate an immune response against intracellular antigens that are otherwise only targetable with engineered TCRs. TCR mimic antibodies can be engineered into alternative formats, such as CARs or bispecific T-cell engagers (BiTEs), which may mediate effective T-cell responses against tumor cells in an HLA-restricted manner. Such strategies were pursued for the NY-ESO-1 CT antigen, demonstrating that HLA-A2/NY-ESO-1 peptide-specific CARs could mediate tumor recognition, which opens up an exciting potential for broadening the repertoire of CAR T-cell targets (43, 44). Recently, a computational transmembrane analysis predicted an extracellular region of the PRAME protein that could be specifically targeted by a conventional PRAME-specific antibody on multiple solid and hematological cancer cell lines *in vitro* and *in vivo*, thereby presenting new opportunities for additional CAR strategies targeting this protein (38). These results, and the fact that PRAME is overexpressed in many malignancies, indicate that PRAME is a promising target for CAR T-cell therapy (45, 46). Other CT antigens with a proposed surface localization include CT83, SP17, SLCO6A1, and PLAC1 (47–50). SP17 is overexpressed in multiple cancer types (48, 51–56), and expression is upregulated by DNMTis (57). SP17 is highly immunogenic (58), but SP17 expression in human ciliated cells of various normal tissues brings into question its suitability as an immunotherapeutic target (51, 59, 60). Nonetheless, SP17 vaccination of humans has been shown to be well-tolerated, with no side-effects regarding expression in normal cells (61). Further investigations must be performed to elucidate the potential of targeting SP17 by immunotherapy. CT83 is also expressed in multiple cancers, such as breast, gastric, and lung cancers, and the expression can be upregulated by DNMTis (62–67). CT83 as a target for TCR-based therapies has shown promising results (64, 68), but the potential of the antigen as a target for CAR T-cell therapy remains unexplored. Similarly, CAR T-cell therapy or alternative antibody therapy has not been pursued for SLO6A1 and PLAC1 despite interesting potential.

CARs with alternative antigen-binding domains are also being investigated to overcome the challenges with target identification. Although not a CT antigen in a strict sense, the IL13-type receptor IL13RA2 is mainly expressed in testis among healthy tissues (69). This receptor recognizes IL13 with higher affinity than the ubiquitously expressed IL13RA1 (69, 70). IL13RA2 is often overexpressed in glioblastoma multiforme (GBM), and expression is correlated with poor patient outcome. New treatment strategies for GBM are much needed, and therefore IL13RA2 is being investigated as a new therapeutic target (71). CARs with antigen-binding domains composed of IL13 mutants, with increased affinity for IL13RA2 and lowered affinity for IL13RA1, have been developed. Preclinical studies investigating these CARs (69, 70, 72), show anti-tumor efficacy and low on-target/off-tumor toxicity after intracranial delivery, due to the abscence of IL13RA2 expression in normal brain tissue. Clinical studies (NCT00730613, NCT01082926, NCT02208362) confirm these promising results and only show manageable side-effects. For example, complete remission was observed in a single patient with recurrent GBM for 7.5 months after several rounds of intracranial delivery of a second-generation IL13RA2 CAR (73). IL13RA2 is also expressed by different immune cells, and IL13RA2 expression in these cells is correlated with immune inhibition. Eradication of cells expressing IL13RA2 by CAR T cells can therefore also increase anti-tumor immunity (69).

## Discussion

To date, the most common immunotherapy is immune checkpoint blockade, which unleashes the activity of T cells by blocking the immune checkpoint molecules PD-1 and CTLA-4 (74). The clinical response to immune checkpoint blockade is generally most significant in patients with tumors that carry a high mutational burden, such as melanoma and non-small-cell lung cancer, but even in these cancer types, the response varies among patients (75). CAR T-cell therapy is an attractive option for non-responders to immune checkpoint blockade and for patients with less immunogenic tumors, such as breast, pancreatic, and some hematological cancers such as AML and ALL (2, 76). However, common obstacles to CAR T-cell therapy must be overcome to ensure an effective clinical response, including the identification of appropriate CAR targets. CT antigens show a restricted expression pattern toward testis and tumor cells, and combining CT antigen-specific CAR T cells with epigenetic therapy, such as DNMTis, can diminish the heterogeneous expression of these antigens within tumors. One might speculate that epigenetic modulators also induce CT antigen expression in healthy tissue to cause serious side effects, but the induction by DNMTis seems to be tumor-specific (29). This may be due to differences in chromatin organization and epigenetic control of gene expression, leaving cancer cells more susceptible to epigenetic enhancement of CT antigen expression than normal cells, but the subject needs further clarification. The safety of combining epigenetic enhancement of antigen presentation with adoptive transfer was further validated in a murine model, where no adverse effects were reported (35).

Even after upregulation of antigen expression by epigenetic modulators, antigen-escape variants, in the form of antigen-negative cells, may be present in tumors. Also, antigen escape can occur as a consequence of the highly selective pressure from mono-specific CAR T cells. Thus, targeting multiple antigens simultaneously may be required for complete responses. Different strategies to achieve this are now being investigated, e.g., pooled uni-specific CAR T cells, bi-specific CAR T cells, and tandem CAR T cells, and are showing promising results in decreasing antigen escape by tumor cells and increasing anti-tumor efficacy (77–80); for example, CAR T-cell therapy using a tandem CAR redirected against both IL13RA2 and HER2 was able to mitigate antigen escape in a murine glioblastoma model compared to uni-specific CARs (81).

Another obstacle to CAR T-cell therapy in regard to solid tumors is the observed low T-cell trafficking to tumor tissue and the hostile TME surrounding the tumor cells. It is now clear that, apart from upregulating CT antigens, DNMTis upregulate a series of immune pathways that augment tumor recognition and elimination by T cells, such as interferon signaling pathways, cytokine and chemokine signaling, inflammation, and genes in the antigen presentation and processing machinery (82–84). DNMTis cause hypomethylation of repeat elements of DNA, leading to increased activation of these regions and increased expression of endogenous retroviral dsRNA in the cytosol. The increased amount of dsRNA in the cytosol triggers a dsRNA sensing pathway as a viral defense mechanism, causing increased release of proinflammatory cytokines and interferons. These molecules act on cells in the nearby environments, leading to inhibition of cellular proliferation and release of chemokines, such as CXCL9/10, that attract cytotoxic T cells to the TME (85, 86). The molecules also have an effect on immune cells in the TME, initiating an innate immune response and increased anti-tumor immunity. Thus, DNMTis may change the hostile TME toward a more T-cell supportive state, which can augment the effect of immunotherapy when used in combination. A side-effect of DNMTis, observed in tumors, is increased expression of PD-L1 and CTLA-4 due to decreased methylation of adjacent promotor regions (87, 88). This increased expression provides the rationale for triple combination therapy of CT antigen-specific CAR T cells, epigenetic drugs, and immune checkpoint blockade.

In conclusion, epigenetic treatment can augment the clinical efficacy of CT antigen-specific CAR T-cell therapy by increasing surface CT antigen expression and diminishing the inhibitory state of the TME, and the therapeutic benefits of combining the two should be pursued through preclinical and clinical testing.

## Author Contributions

MG and MJ contributed equally to the writing of the manuscript. All authors contributed to the article and approved the submitted version.

## Conflict of Interest

The authors declare that the research was conducted in the absence of any commercial or financial relationships that could be construed as a potential conflict of interest.
